# Supplementary material for: Fumonisin Production by Fusarium verticillioides in Maize Genotypes Cultivated in Different Environments
Source: Toxins (Basel). 2019 Apr 10;11(4):215. doi: 10.3390/toxins11040215 (PMC6520941; doi:10.3390/toxins11040215)
Supplement: Supplementary file 1 [file toxins-11-00215-s001.pdf]

toxins

Supplementary Materials: Fumonisin Production by Fusarium verticillioides in Maize Genotypes Cultivated in Different Environments

Oelton Ferreira Rosa Junior, Mateus Sunti Dalcin, Vitor L. Nascimento, Fernando Machado Haesbaert, Talita Pereira de Souza Ferreira, Rodrigo Ribeiro Fidelis, Renato de Almeida Sarmento, Raimundo Wagner de Souza Aguiar, Eugenio Eduardo de Oliveira and Gil Rodrigues dos Santos

Table S1. Analysis of joint variance for a Thousand Kernel Weight (TKW) and disease severity considering two methods of inoculation of *Fusarium verticillioides* in 10 commercial maize hybrids planted at four different localities in Brazil.

	TKW					Disease Severity			
FV	GL	QM	Fc	Ftab	F Test	QM	Fc	Ftab	F Test
BLOCK/ENVIRONMENT	8	753.64	205.67	1.98	*	0.13	1.13	1.98	NS
INOCULATION	2	13290.95	4.98	3.03	*	121.97	41.25	3.10	*
GENOTYPE	9	16437.67	6.16	1.92	*	12.18	4.12	1.99	*
INOCULATION*GENOTYPE	18	863.66	0.32	1.65	NS	1.76	0.60	1.72	NS
ENVIRONMENT	3	184416.19	53.91	2.72	*	69.90	22.72	2.70	*
ENVIRONMENT*TREATMENT1	87	2667.55	727.97	1.33	*	2.96	26.72	1.33	*
MEDIUM ERROR	232	3.66				0.11			
Average: 285.2		CV: 19.82				Average:	6.13	CV:	5.43

* Significant at the 5% level by Test F. 1 Treatment: Combination of factors levels: inoculation and genotype. NS: Not significant.

Table S2. Pearson's linear correlations below the main diagonal and p-value T test for correlation above the main diagonal between the variables Fumonisin B1 (FB1), Fumonisin B2 (FB2), Thousand Kernel Weight (TKW), disease severity (SEV), temperature maximum (°C Max) and minimum temperature (°C Min) of maize hybrids.

30K75Y	FB1	FB2	TKW	SEV.	°C Max	°C Min	Average
FB1	1	0	0	0.017	0.001	0.001	0.001
FB2	0.99*	1	0	0.003	0	0	0
MMG	−0.56*	−0.63NS	1	0	0.228	0.118	0.169
SEV.	0.40*	0.49*	−0.61*	1	0.029	0.004	0.012
°C Max	0.52*	0.56*	−0.21NS	0.36*	1	0	0
°C Min	0.54*	0.58*	−0.27NS	0.47*	0.95*	1	0
Media	0.54	0.57	−0.23	0.42	0.990195745	0.981625806	1
32R48YH							
FB1	1	0	0.098	0	0	0.002	0
FB2	0.99*	1	0.14	0	0	0.004	0
MMG	0.28NS	0.25NS	1	0.003	0.013	0.002	0.005
SEV.	0.73*	0.71*	0.47*	1	0.00	0	0
°C Max	0.70*	0.66*	0.41*	0.75*	1	0	0
°C Min	0.50*	0.47*	0.50*	0.68*	0.95*	1	0
Media	0.61	0.59	0.45	0.728	0.99	0.982	1
DKPDKB240PRO2							
FB1	1	0	0.056	0.066	0.001	0.002	0.001
FB2	0.969	1	0.118	0.193	0.016	0.015	0.014
MMG	−0.32NS	−0.27NS	1	0	0.04	0.006	0.017
SEV.	0.31NS	0.222NS	−0.81*	1	0.005	0.001	0.002
°C Max	0.54*	0.40*	−0.35*	0.46*	1	0	0
°C Min	0.51*	0.40*	−0.45*	0.53*	0.95*	1	0
Media	0.54	0.41	−0.394	0.498	0.99	0.982	1

DKPDKB390PRO2							
FB1	1	0	0.227	0.372	0.006	0.144	0.026
FB2	0.919*	1	0.287	0.158	0.289	0.903	0.572
MMG	−0.21NS	−0.18NS	1	0.654	0.511	0.889	0.658
SEV.	−0.15NS	−0.24NS	−0.08NS	1	0.301	0.032	0.13
°C Max	0.45*	0.18NS	−0.11NS	0.18NS	1	0	0
°C Min	0.25NS	−0.02NS	−0.02NS	0.36*	0.96*	1	0
Media	0.37	0.10	−0.076	0.257	0.99	0.982	1
DOW30A37PW							
FB1	1	0	0	0.002	0.731	0.382	0.565
FB2	0.95*	1	0	0.016	0.222	0.138	0.177
MMG	−0.67*	−0.63*	1	0	0.039	0.029	0.032
SEV.	0.51*	0.399*	−0.60*	1	0.004	0.002	0.003
°C Max	0.06NS	0.21NS	−0.35*	0.463*	1	0	0
°C Min	0.15NS	0.25NS	−0.36*	0.508*	0.945*	1	0
Media	0.10	0.23	−0.358	0.489	0.99	0.982	1
MFRDKB310PRO2							
FB1	1	0	0.022	0	0.463	0.06	0.22
FB2	0.988*	1	0.013	0	0.712	0.112	0.373
MMG	−0.38*	−0.41*	1	0	0.067	0.082	0.069
SEV.	0.75*	0.755*	−0.68*	1	0.607	0.909	0.801
°C Max	0.126NS	0.064NS	0.309NS	−0.09NS	1	0	0
°C Min	0.317NS	0.27NS	0.294NS	0.02NS	0.945NS	1	0
Media	0.21	0.153	0.307	−0.043	0.99	0.982	1
P3250							
FB1	1	0	0.003	0.048	0	0.001	0
FB2	0.976*	1	0.009	0.043	0	0.001	0
MMG	−0.49*	−0.43*	1	0.003	0.884	0.944	0.956
SEV.	0.332*	0.339*	−0.48*	1	0.581	0.461	0.523
°C Max	0.582*	0.637*	−0.03NS	0.095NS	1	0	0
°C Min	0.52*	0.541*	0.012NS	0.127NS	0.945*	1	0
Media	0.563	0.604	−0.009	0.11	0.99	0.982	1
P3340YHR							
FB1	1	0	0.063	0	0	0	0
FB2	0.954	1	0.098	0	0.003	0	0.001
MMG	−0.31NS	−0.28NS	1	0	0.472	0.88	0.628
SEV.	0.606*	0.66*	−0.55*	1	0.53	0.206	0.364
°C Max	0.66*	0.49*	−0.12NS	0.108NS	1	0	0
°C Min	0.76*	0.60*	−0.03NS	0.216NS	0.945*	1	0
Media	0.697	0.544	−0.084	0.156	0.99	0.982	1
P3630H							
FB1	1	0	0.727	0.949	0	0.019	0.001
FB2	1	1	0.718	0.943	0	0.015	0.001
MMG	0.06NS	0.06NS	1	0.001	0.373	0.234	0.303
SEV.	0.01NS	0.01NS	−0.52*	1	0.06	0.026	0.038
°C Max	0.60*	0.603*	−0.15NS	0.32NS	1	0	0
°C Min	0.391*	0.402*	−0.24NS	0.37*	0.96*	1	0
Media	0.514	0.525	−0.177	0.347	0.99	0.982	1
P4285H							
FB1	1	0	0.06	0.008	0.07	0.167	0.099
FB2	0.98*	1	0.023	0.041	0.05	0.13	0.071
MMG	−0.32NS	−0.38*	1	0.026	0.102	0.33	0.171
SEV.	0.44*	0.342*	−0.37*	1	0.017	0.121	0.04
°C Max	0.305*	0.332*	−0.28NS	0.394*	1	0	0
°C Min	0.235NS	0.26NS	−0.17NS	0.26NS	0.945*	1	0
Media	0.279	0.305	−0.233	0.344	0.99	0.982	1
